# Shoulder disorders in female working-age population: a cross sectional study

**DOI:** 10.1186/1471-2474-15-118

**Published:** 2014-04-04

**Authors:** Roberto Meroni, Michele Scelsi, Paola Boria, Valerio Sansone

**Affiliations:** 1 Department of Surgery and Interdisciplinary Medicine, University of Milano-Bicocca, Program in Physical Therapy, Istituti Clinici Zucchi, Piazza Madonnina, 1-20841 Carate Brianza, MB, Italy; 2 Clinica Ortopedica dell’ Università degli Studi di Milano, Milan, Italy; 3 Occupational Medicine, Private Practice, Milan, Italy; 4 Istituto Ortopedico Galeazzi IRCCS, Milan, Italy

**Keywords:** Shoulder, Musculoskeletal disorders, Prevalence, Women, Pain, Clinical examination, Shoulder imaging

## Abstract

**Background:**

Musculoskeletal disorders (MSDs) are among the most common pathologies in the general population. However, research into the prevalence of upper arm MSDs is hampered by a lack of uniformity in case definition, and by the absence of a gold standard for measurement. Furthermore, some sectors of the population have benefited from extensive research whilst others have largely been ignored. Study Design: Cross-sectional study. Objectives: to investigate the prevalence of shoulder MSDs in a working age female population not exposed to specific occupational risk factors such as heavy and/or repetitive work, assessing the differences in prevalence recorded by using three different standard measurement tools.

**Methods:**

302 working aged women were enrolled in this study (age 20–55 years). Each subject underwent three different assessments: standardized questionnaires for symptoms and disability and the SF36 health survey, a clinical assessment performed by a blinded orthopaedic specialist, and an imaging assessment by means of ultrasound (US) and Magnetic Resonance (MR) if indicated.

**Results:**

According to the questionnaire 77 subjects (25.5%) complained of shoulder pain whilst 225 (74.5%) were asymptomatic. According to the clinical examination, 31 subjects (10.3%) resulted positive, whereas 271 subjects (89.7%) had normal shoulders. According to the imaging findings, 26 subjects (8.6%) had alterations to the anatomical structures of the shoulder, whilst 276 subjects (91.4%) had no detectable abnormalities in either shoulder. In all assessments, the prevalence increased with age (p = 0.001).

**Conclusion:**

Depending on the outcome measure used, the prevalence of reported MSDs of the shoulder varies considerably. There is a striking difference between the prevalence of subjective reported symptoms and the standardized clinical/imaging examinations. However, the results of all the assessments did concur in one aspect; there was a significant trend of increased prevalence of shoulder MSDs with age. When looking at reported prevalence, this study shows the importance of noting the measurement method used before making comparisons, as it can vary considerably. The epidemic of shoulder pain reported is not indicative of an epidemic of shoulder pathology.

## Background

Musculoskeletal disorders (MSDs) are among the most common pathologies in the general population. Taken together they represent the largest category of compensable diseases in workers [1-3], with a relevant impact on the individual, their employing organization and the economy in general. It is estimated that in Europe, MSDs pose a financial burden of between 0.5 and 2% of GDP [4]*.* The shoulder and neck are involved in 20 to 30% of the cases [5,6]. However, research into the prevalence of upper arm MSDs is hampered by a lack of uniformity in case definition [7], and by the absence of a gold standard for measurement [8]. Furthermore, some sectors of the population have benefited from extensive research whilst others have largely been ignored [9].

Musculoskeletal disorders (MSDs) is a broad term used in the literature which encompasses a range of degenerative, dysfunctional and inflammatory conditions affecting the locomotor apparatus. With regard to the upper limb, the term MSD is used to cover a range of clearly defined pathologies such as epicondylitis, carpal tunnel syndrome, subacromial impingement etc. as well as non-specific pain syndromes characterized by an unclear clinical behaviour and radiological appearance. Over the years a group of more specific terms such as repetitive strain injury (RSI) [10], cumulative trauma disorders (CTDs), occupational overuse syndrome [11] and work-related upper limb disorders (WR-ULDs) [12] have been adopted, although the terminology varies from study to study [13]. The wide-ranging and often ambiguous definitions of upper limb MSDs, and the variety of criteria used to diagnose them, generate uncertainty in the interpretation of results and in comparison between studies [13]. The absence of a reference standard means data cannot be pooled across industry sectors, geographical areas, demographic groups etc., limiting the ability to evaluate the true social and economic impact of upper limb MSDs [9].

Despite the growing numbers of women reporting upper limb MSDs [9], very little information can be found in the current literature quantifying the prevalence of shoulder pain in working-age female population. Indeed, in their 2010 report, the European Agency for Safety and Health at Work called for more research into upper limb MSDs occurring in higher-risk groups such as women, younger and temporary workers, as these groups have not benefited from specific study [9]. In general, women seem to be particularly at risk with a higher prevalence of upper limb and shoulder MSDs than men [6,14-16]. Possible explanations for the gender difference in prevalence are that male and female workers have different exposure to risk factors, that women have a lower pain threshold [17] and that they might be more prone to express pain and symptoms [18,19]. Another possible hypothesis to explain this apparent disparity is that many of these female patients are suffering from non-specific pain, for which psychological or psychosocial factors have been invoked [20,21].

Given the lack of research into this area, and the recommendations from the European Agency for Safety and Health at Work, we decided to conduct a study into the prevalence of shoulder MSDs in a working age female population not exposed to specific occupational risk factors such as heavy and/or repetitive work, using three different standard measurement tools (questionnaires, orthopaedic clinical examination and imaging) to see if there were any differences in prevalence values observed. In this way, we hoped to create a possible reference for future prevalence studies comparing a “general” population and subjects exposed to specific musculoskeletal risk factors. To our knowledge, this is the first study to use these three tools to measure prevalence in the same female, working-age population.

## Methods

Between January and March 2012 three-hundred and two working-aged women were enrolled in this study. Inclusion criteria were being of age between 20 and 55 years. Exclusion criteria were the presence of evident or previously diagnosed major pathologies such as brachial plexus palsy, neoplasms, rheumatic-linked conditions, previous shoulder trauma and the presence of specific risk factors such as heavy and/or repetitive work. Repetitive work was defined as work that involved continuous repetitive hand or arm movements (e.g. data entry, packing, letter sorting, shop cashier, machine feeding, sewing etc.) [22,23]. The participants of the study were volunteers consecutively enrolled among the customers of three different supermarkets, located in the same province of northern Italy. All subjects were required to sign an informed consent form. A pre-paid gift card was given to reduce possible selection-bias; a free orthopaedic and ultrasonographical examination and the amount of time needed to complete the questionnaires might have been selective factors for subjects with a pre-existing pathology.

The first stage of the study required each subject to respond to a standardized Nordic-style questionnaire that investigated pain of the upper limb [24] (see Additional file 1). An orthopedic specialist administered the questionnaire, to ensure that the subjects fully understood each question. Subjects were asked if they had pain in their shoulders; if the answer was positive, it was noted whether they had taken pharmaceutical drugs, undergone physiotherapy, been to see a specialist or undergone an imaging examination. We chose a simple definition of pain, that has been used elsewhere in the Literature [13], in order to capture chronic, acute or continuous symptoms. “Pain” in our questionnaire was defined as being pain for at least one day a month, or for at least seven consecutive days in the past year. A positive response to either of these possibilities was defined as a “symptomatic shoulder”. A negative response was deemed an “asymptomatic shoulder”. Once this questionnaire had been completed, each subject compiled the SF-36 mental and physical evaluation.

Subjects then passed immediately to the next phase of the study, which consisted of a standardized clinical examination performed by an experienced shoulder orthopaedic specialist. Both shoulders were assessed by measuring the range of motion, performing standard clinical tests (Jobe, Neer, Hawkins-Kennedy [25-27]) and assessing the pull force at 90 degrees of abduction in the scapular plane. From this data the Constant score was calculated for both shoulders [28-30]. If the Constant score was below the normal values for female subjects (specified by Constant [28]) the subject was deemed “abnormal”.

Once the orthopedic specialist examination was completed, a blinded musculoskeletal radiologist (with more than 20 years experience) performed a dynamic ultrasonography of both shoulders in all subjects. (Logiq E9 with a 15 MHz linear probe, *G.E Healthcare*, Milwaukee, WI, USA)*.* In the case of inconclusive findings (based on the radiologist’s judgment the radiologist could complete the evaluation with MRI (Achieva 1.5 T-A, *Philips Healthcare*). Rotator cuff/long head of the biceps tendon lesions, tendon calcifications, bursitis, capsulitis, acromio-clavicular joint arthritis, and all other morphological and degenerative alterations were registered, and the subject was deemed to have an “abnormal” shoulder. Subjects were instructed not to report any complaint during the clinical and screening examinations, since they participated in a blinded protocol.

Data Analysis: descriptive statistics were used to describe the study sample, Pearson correlation was used for investigating the relationship between two quantitative, continuous variables, independent Student’s *t*-test was used to compare the means of two independent samples, one way analysis of variance (ANOVA) was used to test for differences among more than two groups. The chi square statistic was used to investigate whether distributions of categorical variables differ from one another. SPSS Version 19 (Version 19; IBM, Armonk, NY) was used for analysis.

The study was presented to the Institutional Review Board of the Galeazzi Institute, according to our standard procedure. The research protocol was given a detailed examination and evaluation, and permission to proceed was granted since no invasive or potentially dangerous examination was performed on the subjects.

## Results and discussion

### Description of the sample

Anthropometric data are given in Tables 1 and 2. We found a statistically significant positive relationship between both the weight and the age (Pearson correlation: 0.163, p = 0.004) and the weight and the pull force (Pearson correlation: 0.244 for dominant, 0.256 for non dominant, p = 0.001). The differences between subjects with either right or left dominance were not statistically significant.

**Table 1:** Anthropometric data of the cohort

	**MEAN ± SD (range: min-max.)**
**Age (yrs)**	38.5 ± 9.4 (20–55)
**Height (cm)**	163.6 ± 6 (150–180)
**Weight (Kg)**	62.1 ± 12 (40–115)
**BMI (Kg/m**^**2**^**)**	23.2 ± 4.2 (14.5-41.9)
**Pull force dominant arm (Kg)**	6.3 ± 2.4 (3.6-11.8)
**Pull force non-dominant arm (Kg)**	5.8 ± 1.8 (3.1-11.8)
**Right dominance**	93.7%
**Left dominance**	6.3%

**Table 2:** Characteristics of the sample stratified by age

**Age**			**Height cm**	**Weight Kg**	**BMI Kg/m**^**2**^	**Pull force Kg**	
						**Dominant**	**Non-dominant**
**Yrs**	**No.**	**%**	**Mean ± SD**	**Mean ± SD**	**Mean ± SD**	**Mean ± SD**	**Mean ± SD**
**20-24**	22	*7.3*	164.9 ± 5.6	59.2 ± 10.4	21.7 ± 3.35	6.3 ± 1.6	5.7 ± 1.5
**25-29**	36	*11.9*	164.5 ± 5.6	61.0 ± 11.3	22.4 ± 3.44	5.7 ± 1.6	5.3 ± 1.6
**30-34**	46	*15.2*	163.1 ± 5.9	59.7 ± 9.9	22.4 ± 3.56	6.4 ± 1.8	6.2 ± 1.5
**35-39**	61	*20.2*	164.2 ± 6.8	62.4 ± 14.3	23.0 ± 4.55	6.6 ± 1.9	6.1 ± 1.6
**40-44**	43	*14.2*	165.1 ± 5.9	60.3 ± 10.1	22.1 ± 3.73	6.1 ± 2	5.8 ± 1.9
**45-49**	42	*13.9*	163.7 ± 5.1	65.3 ± 11.2	24.4 ± 4.30	6.2 ± 2.5	5.8 ± 2.1
**50-55**	52	*17.2*	160.7 ± 5.9	64.7 ± 13.2	25.0 ± 4.80	6.1 ± 1.9	5.5 ± 1.8

### Subjects self reported status and shoulder symptoms

The results for self-evaluated mental and physical health are given in Table 3. As expected, the older age groups had poorer mean scores for the physical standardized component of the SF36, whilst the scores for the mental component remained fairly stable over time.

**Table 3:** SF36 scores stratified by age

**Age**			**Physical standardized component**	**Mental standardized component**
**Yrs**	**No.**	***%***	**Mean ± SD**	**Mean ± SD**
20-24	22	*7.3*	55.3 ± 5.5	48.7 ± 11.9
25-29	36	*11.9*	54.7 ± 4.8	49.3 ± 7.5
30-34	46	*15.2*	52.3 ± 8.2	46.8 ± 10.8
35-39	61	*20.2*	51.2 ± 9.0	50.2 ± 8.0
40-44	43	*14.2*	51.2 ± 6.0	47.8 ± 8.8
45-49	42	*13.9*	48.4 ± 8.7	49.9 ± 9.5
50-55	52	*17.2*	46.0 ± 10.1	48.2 ± 8.9
**Total**	**302**	***100***	**50.8** ± **8.5**	**48.7** ± **9.2**
Anova between groups for Physical Standardized Component: F = 6.375, p = 0.001*				
Anova between groups for Mental Standardized Component: F = 0.818, p = 0.556				

*= statistically significant.

According to the Nordic-style questionnaire, 77 subjects (25.4%) complained of shoulder pain, of which 39 (12.9%) complained of pain on the dominant side, 17 (5.6%) on the non-dominant side, and 21 (7%) bilaterally. Two hundred and twenty five subjects (74.5%) were asymptomatic. Prevalence of self-reported shoulder pain increased significantly with age (Table 4); indeed, the mean age was greater in subjects who reported pain in their shoulders (44.1 versus 37.6 years, p = 0.001). For the dominant arm there were significant correlations between pain and an increase of BMI (p = 0.006) and of bodyweight (p = 0.009), and with a decrease of pull force (p = 0.015).

**Table 4:** Prevalence of shoulder pain from the Nordic-style questionnaire, stratified by age

**Age**			**Dominant shoulder**				**Non-dominant shoulder**				**Bilateral**	
			**Symptomatic (60)**		**Asymptomatic (242)**		**Symptomatic (38)**		**Asymptomatic (264)**		**Symptomatic (21)**	
**Yrs**	**No.**	***%***	**No.**	***%***	**No.**	***%***	**No.**	***%***	**No.**	***%***	**No.**	***%***
20-24	**22**	*7.3*	**0**	*0*	**22**	*100*	**0**	*0*	**22**	*100*	**0**	*0*
25-29	**36**	*11.9*	**2**	*5.6*	**34**	*94.4*	**1**	*2.8*	**35**	*97.2*	**0**	*0*
30-34	**46**	*15.2*	**8**	*17.4*	**38**	*82.6*	**2**	*4.3*	**44**	*95.7*	**2**	*4.3*
35-39	**61**	*20.2*	**8**	*23.3*	**53**	*76.7*	**6**	*9.8*	**55**	*90.2*	**3**	*4.9*
40-44	**43**	*14.3*	**10**	*31*	**33**	*69*	**14**	*32.6*	**29**	*67.4*	**6**	*13.9*
45-49	**42**	*13.9*	**13**	*36.5*	**29**	*63.5*	**2**	*4.8*	**40**	*95.2*	**2**	*4.7*
50-55	**52**	*17.2*	**19**	*19.9*	**33**	*80.1*	**13**	*25*	**39**	*75*	**8**	*15.3*
			***X***^***2***^ = 24.640; p = 0.001*				***X***^***2***^ = 34.741; p = 0.001*					

*= statistically significant.

Unsurprisingly, the SF-36 Physical Standardized Component was significantly lower in the symptomatic group with respect to the asymptomatic group (mean 45.7 ± 10 versus 52.7 ± 7.1 points, p = 0.001). Ageing was related to a lower score for the SF-36 Physical Standardized Component as expected, whilst the SF-36 Mental Standardized Component was not related to ageing, shoulder symptoms or clinical shoulder abnormality.

### Clinical shoulder abnormality

According to the clinical examination, 31 subjects (10.3%) had an abnormal shoulder, of which 19 (6.3%) were on the dominant side, 6 (2%) on the non-dominant side and 6 (2%) bilaterally, whilst 271 subjects (89.7%) were judged to have normal shoulders. As expected, the prevalence of shoulder abnormality tended to increase with age for both shoulders (Table 5). Our results showed that subjects with abnormal shoulders were older (p = 0.007 dominant, p = 0.001 non-dominant), with higher BMI (p = 0.046 dominant, p = 0.013 non-dominant) and lower pull force (p = 0.002 dominant, p = 0.037 non dominant).

**Table 5:** Prevalence of abnormal shoulders on clinical examination, stratified by age

**Age**			**Dominant shoulder**				**Non-dominant shoulder**				**Bilateral**	
			**Abnormal (26)**		**Normal (276)**		**Abnormal (11)**		**Normal (291)**		**Abnormal (6)**	
**Yrs**	**No.**	*%*	**No.**	*%*	**No.**	*%*	**No.**	*%*	**No.**	*%*	**No.**	*%*
**20-24**	**22**	*7.3*	**0**	*0*	**0**	*0*	**0**	*0*	**22**	*100*	**0**	*0*
**25-29**	**36**	*11.9*	**1**	*2.8*	**35**	*97.2*	**0**	*0*	**36**	*100*	**0**	*0*
**30-34**	**46**	*15.2*	**3**	*6.5*	**43**	*93.5*	**0**	*0*	**46**	*100*	**0**	*0*
**35-39**	**61**	*20.2*	**5**	*8.2*	**56**	*91.8*	**1**	*1.6*	**60**	*98.4*	**1**	*1.6*
**40-44**	**43**	*14.3*	**3**	*7.0*	**40**	*93*	**2**	*4.7*	**41**	*95.3*	**1**	*2.3*
**45-49**	**42**	*13.9*	**7**	*16.7*	**35**	*83.3*	**2**	*4.8*	**40**	*95.2*	**2**	*4.8*
**50-55**	**52**	*17.2*	**7**	*13.5*	**45**	*86.5*	**6**	*11.5*	**46**	*88.5*	**2**	*3.8*
			***X***^***2***^ = 14.141; p =0.028*				***X***^***2***^ = 14.141; p =0.028*					

*= statistically significant.

### Imaging

According to the US and/or MR, 26 subjects (8.6%) had some alterations of the anatomical structures of the shoulder, including calcifications, bursitis, acromio-clavicular arthritis, rotator cuff tears, sub-acromial impingement, of which 8 (2.6%) on the dominant side, 4 (1.3%) on the non-dominant side and 14 (4.6%) bilaterally (Table 6). Both shoulders were normal in two hundred and seventy-six subjects (91.4%). As with the other assessments, the prevalence of anatomical abnormalities tended to increase with age, both for the dominant and the non-dominant arm, and showed a statistically significant correlation (p = 0.001) (Table 7).

**Table 6:** Presence of abnormalities on imaging (US/MR)

	**Abnormality**	**No. of subjects**
**Dominant shoulder**	Alteration of tendon structure	19 supraspinatus, 18 infraspinatus, 5 subscapularis, 2 LHBT
	Tendinopathy	10 supraspinatus, 15 infraspinatus, 4 subscapularis, 1 LHBT
	Partial tendon tear	1 supraspinatus
	Granular calcification	1 supraspinatus, 4 infraspinatus, 1 subscapularis
	Milk calcification	2 infraspinatus
	Linear calcification	12 supraspinatus, 6 infraspinatus, 4 subscapularis
	Humeral head geodes	10 subjects
	Hill Sachs	2 subjects
	AC joint arthrosis	3 subjects
**Non-dominant shoulder**	Alteration of tendon structure	14 supraspinatus, 16 infraspinatus, 6 subscapularis, 1 LHBT
	Tendinopathy	13 supraspinatus, 13 infraspinatus, 5 subscapularis
	Full tendon tear	1 supraspinatus, 1 subscapularis
	Partial tendon tear	1 supraspinatus
	Subluxation	1 subject
	Granular calcification	3 supraspinatus, 4 infraspinatus
	Linear calcification	7 supraspinatus, 7 infraspinatus, 6 subscapularis
	Humeral head geodes	8 subjects
	Hill Sachs	1 subject
	AC joint arthrosis	3 subjects
	Bone spur	1 subject

**Table 7:** Prevalence of US alterations, stratified by age

**Age**			**Dominant arm**				**Non-dominant arm**			
			**Abnormal**		**Normal**		**Abnormal**		**Normal**	
**Yrs**	**No.**	***%***	**No.**	***%***	**No.**	***%***	**No.**	***%***	**No.**	***%***
**20-24**	**22**	*7.3*	**0**	*0*	**22**	*100*	**0**	*0*	**22**	*100*
**25-29**	**36**	*11.9*	**2**	*5.6*	**34**	*94.4*	**0**	*0*	**36**	*100*
**30-34**	**46**	*15.2*	**1**	*2.2*	**45**	*97.8*	**1**	*2.2*	**45**	*97.8*
**35-39**	**61**	*20.2*	**3**	*4.9*	**58**	*95.1*	**1**	*1.6*	**60**	*98.4*
**40-44**	**43**	*14.3*	**1**	*2.3*	**42**	*97.7*	**0**	*0*	**43**	*100*
**45-49**	**42**	*13.9*	**7**	*16.7*	**35**	*83.3*	**6**	*14.3*	**36**	*85.7*
**50-55**	**52**	*17.2*	**8**	*15.4*	**44**	*84.6*	**10**	*19.2*	**42**	*80.8*
			***X***^***2***^ = 16.263; p = 0.012*				***X***^***2***^ = 31.142; p = 0.001*			

*= statistically significant.

We compared the different prevalence levels observed according to the 3 different methodologies (Table 8). For the dominant arm, in 74.4% (224 subjects) all three different examinations were normal, i.e. the subjects did not complain of pain in the dominant shoulder, the orthopedic specialist did not observe a clinical abnormality, and there were no abnormalities on imaging. We can therefore conclude that the absence of pain is a reasonably reliable indicator that pathology is not present. However, in a small number of subjects, an absence of subjective symptoms was associated with abnormalities on US or clinical examination (6.2% dominant, 4.4% non-dominant), which seems to indicate the presence of “silent” pathologies. Some shoulder disorders, such as calcific tendinopathy, may indeed present without symptoms [31], and in fact all the asymptomatic subjects with abnormal imaging results were found to have rotator cuff calcifications.

**Table 8:** Concordance between the different assessment methods, stratified by normal/abnormal imaging (US/MR) findings

		**Dominant arm**		**Non dominant arm**	
		**No.**	**%**	**No.**	**%**
Abnormal imaging findings	Clinically abnormal	4	1.3	3	1.0
	Symptomatic				
	Clinically abnormal	1	0.3	2	0.7
	Asymptomatic				
	Clinically normal	8	2.6	5	1.7
	Symptomatic				
	Clinically normal	9	3.0	8	2.6
	Asymptomatic				
Normal imaging findings	Clinically normal	224	74.2	251	83.1
	Asymptomatic				
	Clinically abnormal	13	4.3	3	1.0
	Symptomatic				
	Clinically normal	35	11.6	27	8.9
	Symptomatic				
	Clinically abnormal	8	2.6	3	1.0
	Asymptomatic				

If, as shown above, the absence of pain seems to be a valid indicator of the subject’s true pathological status, our results show that the reported presence of pain is not as reliable in predicting that shoulder pathology is present. Up to 11.6% of subjects reported shoulder pain without any sign of shoulder abnormality either in the clinical or imaging examinations (Table 8). This finding seems to support the well-documented role of non-specific pain [20,32], which can be defined as the presence of pain without physical signs or any recognizable underlying pathology [20]. Indeed, the prevalence of non-specific pain in general female adult populations has been estimated to be between 2.5% and 13.7% [20,32]. Although relatively little is known about its aetiology [33], it seems likely that psychological or psychosocial factors are involved [20,21].

The occurrence of abnormal findings for all three assessment methods in the same subject was extremely low; 1.3% for the dominant arm and less than 1% in the non-dominant arm. Indeed, in almost a quarter of the population (24.3%), there was a mixture of normal and abnormal results observed for each subject. Such a striking discordance among the three modalities was only partially expected, however this finding may be supported by observations in the clinical setting. In the musculoskeletal field there are well-documented examples where symptoms and imaging are often not correlated (e.g. protrusion or herniation of spinal disks in low back pain free subjects [34]). Another possible reason for this discordance might be the rationale behind the use of a particular methodology; whilst US and orthopaedic clinical tests are considered the standard for instrumental and clinical shoulder examination [35], the focus of these methodologies is to identify the presence of a structural defect. These methodologies do not account for other important parameters such as alterations in shoulder kinematics and associated muscle activity which may be important in patients with a symptomatic shoulder [36]. This clearly represents a limitation of this study and an interesting development for future studies. Finally, in some cases, this apparent discordance could also be due to the abovementioned role of non-specific pain.

According to the results from all three methodologies, ageing had a statistically significant effect on the prevalence of shoulder MSDs, whether reported or objectively diagnosed, which concurs with existing data on the increase of shoulder tendinopathies with age [37,38].

The question arising from these findings is how we should consider the subjects who demonstrated “conflicting” positive results, and which of these three measures should be regarded to have given the “true” prevalence. Although clinical diagnosis is normally made on the basis of all three of these factors, most studies do not consider all the aspects involved in the clinical decision, reporting prevalence based on the results of only one or two of the methodologies. This may, in part, explain the large variances in prevalence of shoulder MSDs that are observed in the Literature. When investigating the prevalence of shoulder MSDs (and indeed, in all MSDs), the results of all three elements (self-reported symptoms, clinical evaluation and imaging) should be evaluated together, if a reliable prevalence is to be obtained.

## Conclusions

Our results suggest that, depending on the outcome measure used, the prevalence of shoulder MSDs changes significantly, although all three methodologies gave concurring results regarding the relationship between MSD prevalence and age.

With this study we tried to addresses a common dilemma in daily rheumatology and orthopedic practice that refers to an assessment of shoulder pain in women of working age and the proper measures of its evaluation. The results show that using only one measurement method when dealing with shoulder pain may lead to incorrect assumptions. We believe that this paper may support clinicians and researchers in understanding the relationships between different assessment methods, also stimulating more research in this field.

## Abbreviations

RSI: Repetitive strain injury; CTD: Cumulative trauma disorders; WR-ULDs: Work-related upper limb disorders; US: Ultrasound; MSD: Musculoskeletal disease; LHBT: Long head of the biceps tendon; AC joint: Acromion-clavicular joint; BMI: Body mass index.

## Competing interests

Each author certifies that they have no commercial associations that might pose a conflict of interest in connection with the submitted article.

## Authors’ contributions

RM: was involved in study conception and design, drafting the manuscript. MS: was involved in drafting the manuscript. PB: Study conception and design. VS: was involved in study conception and design, final approval. All authors read and approve the final manuscript.

## Pre-publication history

The pre-publication history for this paper can be accessed here:

http://www.biomedcentral.com/1471-2474/15/118/prepub

## Supplementary Material

Additional file 1 Upper limb standardized questionnaire. Click here for file
