# Supplementary material for: Shoulder disorders in female working-age population: a cross sectional study
Source: BMC Musculoskelet Disord. 2014 Apr 4;15:118. doi: 10.1186/1471-2474-15-118 (PMC4233642; doi:10.1186/1471-2474-15-118)
Supplement: Additional file 1 — Upper limb standardized questionnaire. [file 1471-2474-15-118-S1.docx]

indicate the areas affected by your symptoms and describe their characteristics


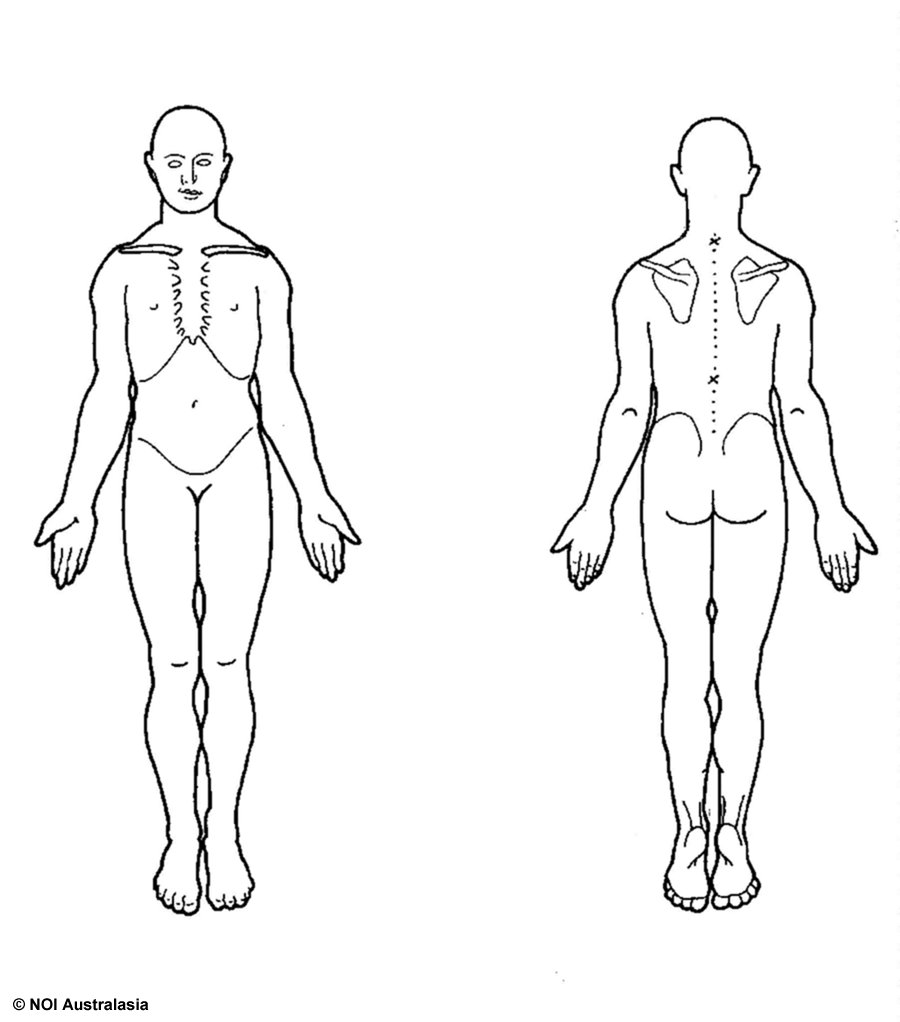


OBSERVATIONS:

____________________________________________________________________________________________

____________________________________________________________________________________________

____________________________________________________________________________________________

____________________________________________________________________________________________

____________________________________________________________________________________________

____________________________________________________________________________________________

____________________________________________________________________________________________

| **PREVIOUS DIAGNOSES** | □ YES □ NO | | |
| --- | --- | --- | --- |
| **SHOULDER** (periarthritis scapolo-humeral; tendonitis etc.) | □ YES □ NO | **WHEN?** |  |
| **WHICH** | | | |
| **ELBOW** (epicondilitis; epitrocleitis; etc.) | □ YES □ NO | **WHEN?** |  |
| **WHICH** | | | |
| **WRIST/HAND:** tendonitis; tendon cysts etc | □ YES □ NO | **WHEN?** |  |
| **WHICH** | | | |
| **WRIST/HAND**: Carpal tunnel syndrome, Guyon | □ YES □ NO | **WHEN?** |  |
| **WHICH** | | | |
| **UPPER LIMB TRAUMA** | | | |
| **SHOULDER** (bruising – fractures – muscle strains etc.) | □ YES □ NO | **WHEN?** |  |
| **WHICH** | | | |
| **ELBOW** (bruising – fractures – muscle strains etc.) | □ YES □ NO | **WHEN?** |  |
| **WHICH** | | | |
| **WRIST/HAND** (bruising – fractures – muscle strains etc.) | □ YES □ NO | **WHEN?** |  |
| **WHICH** | | | |

| SHOULDER PAIN | □ NO □ YES | | **WHEN DID SYMPTOMS FIRST APPEAR? (year)** | |  | R | L |
| --- | --- | --- | --- | --- | --- | --- | --- |
| L  R  | **Related to the symptoms:** | | | PAIN DURING MOVEMENT | | □ | □ |
|  | □ | Taken medication | | PAIN AT REST | | □ | □ |
|  | **Has undergone:** | | | **SIGNIFICANT PAIN** | | R | L |
|  | □ | Physiotherapy | | continuous pain | | □ | □ |
|  | □ | GP/Orthopaedic visit | | pain at least 1 week in the last year | | □ | □ |
|  | □ | X-ray | | Pain at least once a month in the last year | | □ | □ |
|  | □ | US/MRI | | **NON-SIGNIFICANT PAIN** | | R | L |
|  |  |  | | less frequent pain | | □ | □ |
| ELBOW PAIN | □ NO □ YES | | **WHEN DID SYMPTOMS FIRST APPEAR? (year)** | |  | R | L |
| L  R  | **Related to the symptoms:** | | | PAIN DURING MOVEMENT | | □ | □ |
|  | □ | Taken medication | | PAIN AT REST | | □ | □ |
|  | **Has undergone:** | | | **SIGNIFICANT PAIN** | | R | L |
|  | □ | Physiotherapy | | continuous pain | | □ | □ |
|  | □ | GP/Orthopaedic visit | | pain at least 1 week in the last year | | □ | □ |
|  | □ | X-RAY | | Pain at least once a month in the last year | | □ | □ |
|  | □ | US/MRI | | **NON-SIGNIFICANT PAIN** | | R | L |
|  | □ | EMG (electromyography) | | less frequent pain | | □ | □ |
| HAND/WRIST PAIN | □ NO □ YES | | **WHEN DID SYMPTOMS FIRST APPEAR? (year)** | |  | R | L |
|  | **Related to the symptoms:** | | | PAIN GRIPPING | | □ | □ |
|  | □ | Taken medication | | PAIN DURING MOVEMENT | | □ | □ |
|  |  |  | | PAIN AT REST | | □ | □ |
|  |  |  | | PAIN IN THUMB | | □ | □ |
|  |  |  | | PAIN IN OTHER FINGERS | | □ | □ |
|  |  |  | | PAIN IN THE PALM | | □ | □ |
|  |  |  | | WRIST PAIN | | □ | □ |
|  | **Has undergone:** | | | **SIGNIFICANT PAIN** | | R | L |
|  | □ | Physiotherapy | | continuous pain | | □ | □ |
|  | □ | GP/Orthopaedic visit | | pain at least 1 week in the last year | | □ | □ |
|  | □ | X-RAY | | Pain at least once a month in the last year | | □ | □ |
|  | □ | US/MRI | | **NON-SIGNIFICANT PAIN** | | R | L |
| indicate the areas affected | □ | EMG (electromyography) | | less frequent pain | | □ | □ |

NB: in the diagnosis of significant pain/symptoms IN THE UPPER LIMBS, YES indicates the presence of: continuous pain, or pain at least 1 one week in the last 12 months, or pain at least once a month in the last year

| NIGHTIME PARESTHESIA | □ NO □ YES | | **WHEN DID SYMPTOMS FIRST APPEAR? (year)** | |  | R | L |
| --- | --- | --- | --- | --- | --- | --- | --- |
|  | **Related to the symptoms:** | | | ARM | | □ | □ |
|  | □ | Taken medication | | FOREARM | | □ | □ |
|  |  | | | HAND | | □ | □ |
|  |  |  |  | LASTING LESS THAN 10 MINUTES | | □ | □ |
|  |  |  |  | LASTING MORE THAN 10 MINUTES | | □ | □ |
|  |  |  |  | APPEAR WHILE SLEEPING | | □ | □ |
|  |  |  |  | APPEAR WHEN WAKING UP | | □ | □ |
|  | **Has undergone:** | | | **SIGNIFICANT PAIN** | | R | L |
|  | □ | Physiotherapy | | almost every night | | □ | □ |
|  | □ | GP/Orthopaedic visit | | at least one week in the last year | | □ | □ |
|  | □ | X-RAY | | at least one day a month | | □ | □ |
|  | □ | US/MRI | | **NON-SIGNIFICANT PAIN** | | R | L |
| indicate the areas affected | □ | EMG (elettromiografia) | | less frequent symptoms | | □ | □ |
| DAYTIME PARESTHESIA | □ NO □ YES | | **WHEN DID SYMPTOMS FIRST APPEAR? (year)** | |  | R | L |
| L  R  | **Related to the symptoms:** | | | ARM | | □ | □ |
|  | □ | Taken medication | | FOREARM | | □ | □ |
|  |  | | | HAND | | □ | □ |
|  |  |  |  | LASTING LESS THAN 10 MINUTES | | □ | □ |
|  |  |  |  | LASTING MORE THAN 10 MINUTES | | □ | □ |
|  |  |  |  | symptoms when arms are raised | | □ | □ |
|  |  |  |  | symptoms when leaning on the elbow | | □ | □ |
|  |  |  |  | symptoms when gripping or working | | □ | □ |
|  | **Has undergone:** | | | **SIGNIFICANT PAIN** | | R | L |
|  | □ | Physiotherapy | | almost every day | | □ | □ |
|  | □ | GP/Orthopaedic visit | | at least one week in the last year | | □ | □ |
|  | □ | X-RAY | | at least one day a month | | □ | □ |
|  | □ | US/MRI | | **NON-SIGNIFICANT PAIN** | | R | L |
| indicate the areas affected | □ | EMG (electromyography) | | less frequent symptoms | | □ | □ |

| SICK LEAVE TAKEN FOR UPPER LIMB PAIN | □ NO □ YES | IF YES, DAYS: |  |
| --- | --- | --- | --- |

**Part 2 SPINE – SYMPTOMS IN THE LAST 12 MONTHS**

| Mark on the diagram areas of symptoms and any radiation | **CERVICAL SPINE** WHEN DID SYMPTOMS COMMENCE? (year) | | | | |  | |
| --- | --- | --- | --- | --- | --- | --- | --- |
|  | RARELY | | AT LEAST 3-4 EPISODES OF 2-3 DAYS | AT LEAST 3-4 EPISODES, MEDICATION OR TREATMENT REQUIRED | | ALMOST EVERY DAY | |
|  | □ SLIGHT PAIN | | □ SLIGHT PAIN | □ SLIGHT PAIN | | □ SLIGHT PAIN (*) | |
|  | □ PAIN | | □ PAIN (*) | □ PAIN (*) | | □ PAIN (*) | |
| RADIATION TO UPPER LIMB | | | □ NO | □ R □ L | |  | |
| SICK LEAVE TAKEN FOR CERVICAL SPINE SYMPTOMS | | | □ YES (days) |  | |  | |
|  | □ SIGNIFICANT PAIN (*) | | | □ NON-SIGNIFICANT PAIN | | | |
| Mark on the diagram areas of symptoms and any radiation | | **DORSAL** WHEN DID SYMPTOMS COMMENCE? (year) | | | | |  |
|  | | RARELY | | AT LEAST 3-4 EPISODES OF 2-3 DAYS | AT LEAST 3-4 EPISODES, MEDICATION OR TREATMENT REQUIRED | | ALMOST EVERY DAY |
|  |  | □ SLIGHT PAIN | | □ SLIGHT PAIN | □ SLIGHT PAIN | | □ SLIGHT PAIN(*) |
|  |  | □ PAIN | | □ PAIN (*) | □ PAIN (*) | | □ PAIN (*) |
| RADIATION TO HEMITHORAX | | | | □ NO | □ R □ L | |  |
| SICK LEAVE TAKEN FOR DORSAL SPINE SYMPTOMS | | | | □ YES (days) |  | |  |
|  | | □ SIGNIFICANT PAIN (*) | | | □ NON-SIGNIFICANT PAIN | | |

| Mark on the diagram areas of symptoms and any radiation | **LUMBOSACRAL** WHEN DID SYMPTOMS COMMENCE? (year) | | |  |
| --- | --- | --- | --- | --- |
|  | RARELY | AT LEAST 3-4 EPISODES OF 2-3 DAYS | AT LEAST 3-4 EPISODES, MEDICATION OR TREATMENT REQUIRED | ALMOST EVERY DAY |
|  | □ SLIGHT PAIN | □ SLIGHT PAIN | □ SLIGHT PAIN | □ SLIGHT PAIN(*) |
|  | □ PAIN | □ PAIN (*) | □ PAIN (*) | □ PAIN (*) |
| RADIATION TO LOWER LIMBS | | □ NO | □ R □ L |  |
| SICK LEAVE TAKEN FOR LUMBOSACRAL SYMPTOMS | | □ YES (days) |  |  |
|  | □ SIGNIFICANT PAIN (*) | | □ NON-SIGNIFICANT PAIN | |

NB*: in the diagnosis of significant pain/symptoms IN THE SPINE, YES indicates the presence of: PAIN/ SLIGHT PAIN almost every day in the last year, or episodes of pain (3-4 episodes of 2-3 days; 10 episodes of 1 day; 8 episodes of 2 days; 2 episodes of 30 days; 1 episode of 90 days).

| ACUTE EPISODES OF BACK PAIN | | | | | | |
| --- | --- | --- | --- | --- | --- | --- |
| TOTAL N° OF ACUTE EPISODES |  |  | | |  | |
| N° OF ACUTE EPISODES IN LAST YEAR | □ BACK PAIN | |  | □ LUMBOSCIATICA | |  |
| YEAR OF 1ST EPISODE |  | | |  | | |

For acute back pain YES indicates: a period of intense lumbosacral PAIN that prevents bending, straightening, flexing or rotation, the arrival of which may be sudden or gradual, and which lasts at least 2 days, or which requires medication

| **PREVIOUS DIAGNOSES** | | □ YES □ NO | | |
| --- | --- | --- | --- | --- |
| **LUMBOSACRAL HERNIA** | | □ YES □ NO | **WHEN?** |  |
| **LUMBOSACRAL HERNIA, SURGICALLY TREATED** | | □ YES □ NO | **WHEN?** |  |
| **PATHOLOGIES/TRAUMA CERVICAL SPINE** | | □ YES □ NO | **WHEN?** |  |
| **WHICH** |  | | | |
| **PATHOLOGIES/TRAUMA DORSAL SPINE** | | □ YES □ NO | **WHEN?** |  |
| **WHICH** |  | | | |
| **PATHOLOGIES/TRAUMA LUMBOSACRAL SPINE** | | □ YES □ NO | **WHEN?** |  |
| **WHICH** |  | | | |
